# American Medical Association

**Published:** 1860-07

**Authors:** J. Solis Cohen


### EDITORIAL AND MISCELLANEOUS.

### American Medical Association.

*Thirteenth Annual Meeting, Held at New Haven, June 5th, 6th and 7th,* 1860.

Reported expressly for the Medical and Surgical Reporter, by Dr. J. SOLIS COHEN.

FIRST DAY'S PROCEEDINGS—*Morning Session.*

The members of the Association convened at the Chapel of Yale College, and at 11 A. M., were called to order by the President, Henry Miller, M. D., of Kentucky.

A prayer was offered by Prof. Fisher, of Yale College.

Dr. Jonathan Knight, of Connecticut, on behalf of the Committee of Reception, welcomed the members of the Association to the hospitalities of the city. He spoke in graphic terms of the benefits accruing to the profession and to the world by the annual gatherings of the Association, which kept up a good feeling between distant members of the profession ; he recommended such modes of action as would advance the general interests of the profession. He gave an epitome of the gradual progress of medicine, and dwelt at length upon the improvements in surgery, made during the present century, alluding to the operation of lithotripsy, the ligation of the large arteries, and the introduction of anæsthetic agents. The address was listened to with profound attention, and greeted with marked applause.

Dr. Chas. Hooker, Professor of Anatomy and Physiology in the medical department of Yale College, as Chairman of the Committee of Arrangements, addressed the Association as follows:

"*Mr. President and Gentlemen of the American Medical Association :* It is with unwonted gratification that the Committee of Arrangements welcome you to the City of New Haven. And we only bespeak the common feeling of our citizens in saying that we are delighted—nay, proud—to receive you as our guests. We feel that any city is highly honored to become the chosen place of meeting of the American Medical Association—a select delegated national Congress, representative of forty thousand members of a learned and humane profession. As a city, we appreciate this honor, and should be ungrateful did we not receive you with a generous and cordial welcome. You meet, gentlemen, for a great and noble object—for the promotion of science, vitally linked with the interests of humanity. Your meetings have a most happy influence in strengthening those ties by which the great Fraternity of Medicine are bound in social compact. Another salutary incidental benefit of your meetings, results from their affording an annual period for relaxation and social enjoyment.

Too many physicians prematurely break down in their career of usefulness, in consequence of unremitted and arduous application to their professional duties; and many of you now present, whose exhausted physical and mental energies need recruiting, could hardly have been drawn away from your routine of toil and care, but for your sense of bounden duty to aid in the great object of this Association. We congratulate you, therefore, brethren, on this annual recurrence of our National Medical Jubilee. In behalf of the Faculty of Yale, we welcome you to the halls of this ancient seat of learning, in which you are invited to hold your sessions; and in behalf of the citizens, generally, of New Haven, we tender you the hospitalities of our city.

We hope that to all of you this meeting will be a season of pleasant social intercourse long to be remembered for the many friendships here formed ; and we trust that the harmony and wisdom of your counsels will efficiently promote the great benevolent objects of our organization."

He then gave notice to the members that arrangements had been made to accommodate the meeting of the different sections as follows:

On Anatomy and Physiology—in Prof. Woolsey's lecture room.

On Surgery—in the Geological Cabinet.

On Practical Medicine and Obstetrics—in the Geological Cabinet.

On Chemistry and Materia Medica—in the Chemical Laboratory.

On Meteorology, Medical Topography, Epidemic Diseases, Medical Jurisprudence and Hygiene—in the Chemical Laboratory.

At the last annual meeting in Louisville, it had been recommended to divide the Association into the above sections, in order to facilitate the transaction of business.

After the calling of the roll by the Secretary, Dr. S. M. Bemiss, Ky., on motion of Dr. John Atlee, all the medical officers of the Army or Navy, present, were invited to take seats in the Association.

The committee appointed at the last meeting to prepare a code of parliamentary rules for the government of the Association, stated that their report, chiefly the work of Dr. Chas. A. Lindsley, of Conn., was ready, and as it was brief, arrangements had been made for its immediate commitment to the press and distribution among the members, if adopted by the Association.

After some debate, the report was laid on the table.

A recess was here allowed, to permit the delegates from the different States, to choose their respective member of the Committee on Nominations.

At a quarter before one, the Association was again called to order, and the following gentlemen declared the Committee on Nominations:

District of Columbia—Dr. Boyle.
Maryland—C. C. Cox.
Kentucky—R. I. Breckenridge.
North Carolina—James H. Dixon.
Tennessee—I. S. White.
Delaware—Lewis P. Bush.
Louisiana—Austin Flint, Jr.
Minnesota—D. W. Hand.

Georgia—H. W. Brown.
Massachusetts—D. Humphreys Storer.
Maine—Amos Nourse.
Indiana—Daniel Meeker.
New Jersey—J. S. English.
Rhode Island—James H. Eldridge.
New Hampshire—George H. Hubbard.
Illinois—A. S. McArthur.
Mississippi—U. G. Williams.
Michigan—C. L. Ford.
Pennsylvania—Wilson Jewell.
Iowa—D. L. McGugin.
Ohio—Robert Thompson.
Missouri—M. A. Pallen.
Vermont—Charles L. Allen.
Virginia—James H. Conway.
Connecticut—L. N. Beardsley.
South Carolina—H. R. Frost.
New York—H. D. Bulkley.

A motion was passed to invite the members of the Legislature of Connecticut, then in Session at New Haven, to be present at the Afternoon Session, as the address of the retiring President, which would then be delivered, would contain points of medico-legal interest. On motion the Association adjourned to meet again at 3 P. M.

*Afternoon Session.*

At 3 P. M., the Association was called to order by Vice President, H. F. Askew, of Delaware.

Gov. Buckingham and Lieut. Gov. Catlin, of Conn., on the platform, were introduced to the meeting by the chairman.

The retiring President, Dr. Henry Miller, of Ky., then read his valedictory address.

Our space does not permit us to give this address in full. We subjoin a few extracts.

"Gentlemen of the American Medical Association: It affords me a great satisfaction to greet you as representatives of the American medical profession, in this beautiful city—the city of Yale College—a city whose intelligent legislation has made ample provision for the education of her children. Let us accept our annual meeting on this classic ground, as a token that our deliberations shall favor medical education, the improvement of which was the fundamental design of our National Association. In order that this great object should be readily obtained, I need not remind you how necessary a spirit of moderation is to all our discussions, and most fervently do I hope such a spirit prevails, to preside over all our sessions.

The duties of the President of this Association, are exclusively parliamentary. He is not even empowered to fill vacancies in subordinate committees, much less to give information to the Association of the state of the profession, which, it may be resumed, has occupied his thoughts. During my term of office I have been obliged to assume the power of filling vacancies; and in the performance of this, my last official act, I shall take the liberty of adverting to topics of high concern, not only to our profession, but to the public at large.

"At the last meeting of the Association a Committee on Criminal Abortion made their report, which was received and referred to the Committee of Publication. The resolutions appended to the report were adopted, and the President and Secretary authorized to bring this important subject, in the form of a memorial, before Congress, and the several legislatures in the different States of the Union. By reference to the proceedings of the last annual meeting, it will be seen that the committee were requested to continue their labors, and take such measures as were necessary to carry into effect the spirit of the resolutions. The chairman opened a correspondence with me early last winter, offering to place at my disposal extra copies of the report, and also various papers published by him in the *N. A. Medical and Chirugical Review*, containing all the matter necessary for the several legislatures to know, to act properly in the premises. I am happy to acknowledge my obligations for this gentleman's valuable assistance, not only in furnishing the documents he did, but in preparing a memorial, and an address to the State Medical Societies, asking their co-operation to bring this matter before the legislatures of their respective States. The memorial was transmitted, in January last, to the President of the United States and to the Governors of each of the States and Territories of the Union, the legislatures of several of the States being at the time in session. Of the disposition which has been made of these addresses I am not informed, but may indulge the hope that their Excellencies have submitted them to their various legislatures, or will embrace the earliest opportunity of doing so. From want of knowledge of the address of all the State Medical Societies, I was forced, in some instances, to direct to prominent medical gentlemen in various States, through whom I hope the papers reached their destination. I would here recommend that measures be taken by this Association to obtain annually a list of the various medical societies, and the address of their officers, to be published in our transactions. Besides permitting facility of correspondence, this would tend to bind our medical organizations more closely together, and render them more effective in the great work which it is proposed to accomplish through their instrumentality.

Having laid before you, gentlemen, the measures which have been taken to carry into effect the resolutions adopted at the last meeting of the Association on this interesting subject, which involves the honor of our profession and the great interests of Society, it becomes my duty to warn you that obstructions may be thrown in your path, which may require years of ceaseless vigilance and unremitting effort to overcome the popular ignorance on this subject, while many in your own ranks are to be watched. Popular sentiment either winks at abortion in the earlier periods of fœtal development, or only admits it as an indiscretion on the part of the mother, or rashness on the part of the practitioner. This opinion is known to be based upon erroneous views of physiology, derived, doubtless, as all such errors are, from the false speculations of physiologists and naturalists of a former age. Would a pure, delicate minded mother, who would shrink from the very idea of committing an immoral act, relieve herself of that which would otherwise become a burden upon her, were she informed by the physician of the enormity of the crime? The truth should be generally promulgated, that from the moment of conception, the new being, microscopic though it be, is endowed with all that appertains to the development of man. It is at once an individual being, and no more a part of the mother, though she bears it within her, than it is of the father; and is no more dependent on her during its development in the womb, than after birth, when it draws its sustenance from her breast.—Were this taught universally, no woman, however degraded or fallen into vice, would go so far as to permit the destruction of her offspring.

It is difficult for legislation in a free country, where the people are the source of all political power, to rise higher than popular sentiment and intellignce. But it is the duty of all our legislatures, in questions which can only be determined by the science of medical jurisprudence, to endeavor to elevate popular sentiment and remove the ignorance upon this point, rather than to degrade themselves. The necessity of legislation upon this point has been clearly pointed out by the chairman of the committee, and plausible suggestions for the enactment of a suitable law have been prepared by him. We hope the appeal made will not have been in vain.

The subject of medical education has occupied a large share of the attention of the American Medical Association since its first organization. It was indeed the urgent necessity of reform in the administration of its important interest to the profession which called forth from the Medical Society of the State of New York, a design for a National Medical Association, composed of delegates from the different medical societies and colleges, to meet in New York in May, 1846, and afterwards adjourned to meet in Philadelphia, in May, 1847, where it was resolved into the American Medical Association, which has since been in vigorous existence. Unfortunately there have been differences of opinion on the points of medical education, between those of our profession who occupy the position of professors and those who do not, and I may claim to be a mediator between them, as I have been with them, man and boy, for upwards of forty years, engaged in the study and practice of medicine, and for twenty-one years professor in a school, which at one time ranked third in the United States, in regard to the number of pupils. Having laid aside the professional garb two years ago, and become again one "among the herd," I am qualified to act as a "go-between."

The system of medical education adopted in the United States, is derived from our British forefathers, and recognizes the right of the great body of the medical profession to preside over the initiation of candidates for its honors and emoluments.

On the continent of Europe young men are transferred from the gymnasiums to the academies to receive their first lessons, as well as the finishing instruction in the profession of their choice. The different effect of this system from our own is calculated to do injustice to our institutions. The medical schools of this country have always, at least in theory, admitted that it is at once the high privilege and duty of all regular members of the great medical body to receive properly qualified and educated young men into their offices as students, to prescribe their studies, and to prepare them to profit in the largest manner, by the advantages they can nowhere obtain so well as in a properly regulated medical school. The founders of our first medical school were those who first learned from private instruction before they went abroad to the schools.

Here the history of several distinguished medical men, who received their first medical education in the private office of a practitioner, was alluded to.

These men established on this side of the Atlantic an institution in which the American education by private tuition might receive its finishing touch and reward, without the risk of the dangers incident to travel in the old world.

Previous to the establishment of a medical school in Philadelphia, most of the students in medicine received their education in the shops of practitioners, where medical lore was imparted,and the practical part of the apothecary carried on; and after the school was started, a Philadelphia graduate was looked upon as something superior, and more reliable than the less favored multitude of physicians.

If, according to our American system, medical education has its beginning in private tuition, and is completed in medical schools, it follows necessarily that it is the function of the schools to receive their students at the hands of private teachers engaged in the practice of their profession, and to require vouchers that they have gone through the proper course of necessary training.

To dispense with this preparatory course would be a violation of the contract between public and private individuals, and also be an egregious wrong to the students themselves, by an attempt to instil into their minds an instruction which, by previous culture, they are not prepared to receive.

The true distinctions between physicians are not between professors and laymen, but between public and private teachers of medicine. Were medical instruction among us entirely committed to the schools, as in most European countries, this distinction would not exist.

Dr. Miller went on to say, that enough precaution was not taken even in selecting professors; that many teachers were unfit for their position, and their examination of students was a farce. The qualifications of professors ought to be insisted on as well as the qualifications of students.

He spoke of the practical essays of Dr. on medical education, published in in which he complained of the necessity of a higher standard of medical excellence and the extraordinary increase in the number of medical colleges in the United States, and stated that one cause is a want of due care in the selection of professors, by which, at present the standard of excellence is such as to bring into responsible positions those of even less than mediocrity in medical lore and literature.

The necessity of reform in the selection of teachers and the examinations of candidates, was fully insisted on.

The Secretary, Dr. Bemiss, of Ky., then read additional names of delegates whose credentials had been examined by the Committee on Credentials since the morning meeting.

The Nominating Committee here reported the following names as those of officers for the ensuing year. The gentlemen nominated were elected by acclamation:

President—Eli Ives, Conn.

Vice-Presidents—Wilson Jewell, Pa., A. B. Palmer, Mich., Jos. P. Logan, Ga., Jos. N. McDowell, Mo.

Treasurer—Caspar Wistar, Pa.

The Chairman then appointed the following escorts to the officers elect:

For Escort to President—Jonathan Knight, Conn., Dixi Crosby, N. H.

For Escort to Vice-Presidents—W. C. Snead, Ky., Wm. Brodie, Mich., Edward Warren, Md., R. C. Foster, Tenn., K. J. Bowditch, Mass., Lewis A. Sayre, N. Y., John L. Atlee, Pa., Austin Flint, Jr., La.

A number of communications were then read, inviting the members of the association to visit the carriage factories of Messrs. J. & D. Cook & Son, and Messrs. Laurence, Bradley & Pardee, and also an invitation from Wm. H. Russel, M. D., to visit the evening drill and parade of the students in his Collegiate and Commercial Institute.

These were accepted, and hours set apart in which the members could visit the above places without interfering with attendance on the meetings of the Association.

Business was then suspended while the officers elect were conducted to their places by the Committees of Escort, and severally introduced to the Association by the Chairman, Dr. H. F. Askew, of Del.

Dr. Eli Ives, President, in taking his seat, made a short address, in which he stated that, in giving thanks for the honor conferred upon him, he would be ungrateful not to publicly thank the profession for all he was and all he possessed. All his property and reputation had been derived from his profession, and therefore deserved his thanks. His father had been a physician, and one of the founders of medical societies; he had two sons and one grandson, physicians. He had always loved and enjoyed his profession, and to all present he would repeat what Dr. Rush remarked to his class in 1801-2: "Gentlemen, if you don't like your profession, the sooner you leave the better." He had been practicing medicine longer than any present, and that when he could no longer do so, he would himself be carried to the bedside.

Dr. Wilson Jewell, of Pa., stated that it is not customary for Vice-Presidents to make addresses on their election, or to preside over the deliberations of the electing body. The first he would not attempt, but at the request of the President (who is too aged and feeble to preside) he would attempt the latter, and all he asked was allowance for any infirmities. He would endeavor to preside with strict impartiality.

Dr. N. S. Davis, of Ill., introduced the following resolutions to carry into effect the arrangement adopted at the last meeting of the Association, to facilitate the transaction of buisness in the consideration of scientific matters by the division into sections:

*Resolved*, That the general meetings of the Association, after this day, shall be restricted to the morning sessions, and the afternoon sessions commencing at 3 o'clock, shall be devoted to the hearing of papers and discussions in the several sections.

*Resolved*, That each section shall choose its own officers, and make its own rules of order.

*Resolved*, That all essays, voluntary communications and reports, except those from the officers of the Association, and the committees on medical education, medical literature, and the committees on prize essays, shall be first presented or referred to the appropriate section and receive its recommendation, before they can be referred to the committee on publication. The first and second resolutions were adopted. The reading of the third resolution called forth considerable discussion, during which several amendments were proposed and withdrawn. Some members thought that everything appearing in the printed account of the Transactions, should undergo proper revision and alteration by a special committee; others were of opinion that the Association was not responsible, as an association, for every thing that emanated from them, and therefore addresses and papers should appear in the orginal language of their authors without alteration. Several gentlemen refused to submit their papers to any revision, and stated that if the Association did not choose to print them as they were, they would publish them themselves at their own expense. Over an hour was consumed in this discussion, when, on motion, the resolution was laid on the table to give the mover an opportunity of so altering it as to meet the views expressed by the different members.

On the recommendation of Drs. Timothy Child, and David S. Conant, Dr. Wm. B. Little, of San Francisco, Cal., was admitted a member by invitation, there being no representative from his section of the country, a member of the Association. The following gentlemen were appointed a Committee on Voluntary Communications:

E. D. Force, Ky., Thos. W. Blatchford, N. Y., N. D. Davis, Ill., —— ———, Rochester N. Y., Dr. Reuchenberger, Philadelphia.

The Treasurer, Caspar Wister, M. D., of Pa., then made his report:—

He reported that out of a list of 2,000 names, he had only some 200 annual subscribers to the volume of Transactions, and these were obtained only after the persistent solicitations of the Treasurer, addressed to each permanent member, announcing the publication of the last volume of the Transactions. Of the printed volumes of Transactions, there were still on hand, for sale at the following prices: years 1856 and 7, of the organization of the Association, 50 cents each.

Vols. 1, 2, 3, and 4, out of print.

Vols. 5, 7, 8, 9, collectively as a set, $5 00, separately $2 00 each.

Vols. 6, 10, 11, at $2 00 a volume.

Vol. 12, at $3 00.

Cash on hand, April 15, 1859,..................$651 00.
Received from delegates for last volume of Transactions,....................................2,430 00
On hand......................................597 61

The report was accepted, and referred to Committee on Rublication.

On behalf of the Committee on Publication, Dr. Caspar Wister reported that the delay in the appearance of the last volume of the Transactions had been caused by the delay of the authors of several articles in returning corrected proofs of their articles, the proofs having in one instance been detained 9 weeks, and in another 14 weeks. The committee hoped some action would be taken to prevent a similar occurrence in the future. The cost of printing the last volume of Transactions had been $1,659 00.

There were remaining on hand, of the volumes containing the proceedings of the first meeting of the Association, 45 copies; vol. 5,241 copies; vol. 6, 11 copies; vol. 7, 51 copies; vol. 8, 212 copies; vol. 9, 242 copies; vol. 10, 165 copies; vol. 11, 152 copies; vol. 12, 497 copies.

The report was received, and referred to the Committee on Publication.

No other committees being prepared to report, the Association, on motion, adjourned.

[To be Continued.]

